# Post-prison Employment Quality and Future Criminal Justice Contact

**DOI:** 10.7758/rsf.2020.6.1.07

**Published:** 2020-03

**Authors:** JOE LABRIOLA

**Affiliations:** PhD candidate in sociology at the University of California, Berkeley.

**Keywords:** employment, job quality, recidivism

## Abstract

Several theories linking post-prison employment to recidivism suggest that the quality of employment has a causal effect on future criminal justice contact. However, previous work testing these theories has not accounted for differential selection into high-quality employment. Using six years of post-release employment records, I document how post-prison job quality varies by industry. Then, I use inverse propensity score weighting to estimate the effect of job quality on future arrests and prison spells. Some evidence indicates that parolees who find high-quality employment experience fewer arrests or returns to prison than otherwise similar parolees who find low-quality employment, with the effects most evident when comparing employment in the highest- and lowest-quality industries. Low-quality employment does not appear to reduce future criminal justice contact relative to unemployment.

More than 625,000 prisoners were released from state and federal prisons in the United States in 2016 (Carson 2018). A large majority of such released prisoners experience criminal justice contact in the years after release (Alper, Durose, and Markman 2018). Given that extended contact with the criminal justice system is associated with negative effects on employment (Pager 2003), health (Massoglia and Pridemore 2015), and wealth (Harris, Evans, and Beckett 2010), as well as increased disadvantage for children of those experiencing criminal justice contact (Wildeman 2008), it is important to understand the factors that reduce future criminal justice contact.

Sociologists and criminologists emphasize the role of employment in reducing future criminal justice contact after release from prison. Employment has been theorized to reduce economic motivations for crime (Becker 1968; Freeman 1999), facilitate the achievement of normative societal goals (Merton 1938; Agnew 1985), act as an informal social control on parolees (Toby 1957; Sampson and Laub 1993), and provide a routine set of obligations that replace previous criminal activities (Cohen and Felson 1979). In particular, post-prison employment that pays well, is stable, and allows for future earnings growth is thought to be especially important in preventing future criminal justice contact.

However, previous research investigating the connection between post-prison employment quality and recidivism or other forms of criminal justice contact in the United States (Uggen 1999) has failed to adequately control for selection into employment quality, not just employment. This may pose a problem if those who find high-quality post-prison employment differ from those who find low-quality employment in dimensions that are also predictive of future criminal justice contact, such as age, human capital, or prior measures of criminal or antisocial behavior.

In this article, I estimate whether individuals who are first employed after prison in industries that offer relatively high-quality employment are significantly less likely than those who are first employed after prison in industries that offer relatively low-quality employment to be arrested or return to prison in the two years following the beginning of employment. I do so using comprehensive labor market information collected on all prisoners paroled in the state of Michigan in 2003 for six years after the quarter of release from prison, alongside an array of rich demographic, human capital, and criminal justice-related measures. I first measure employment quality within industries along four objective dimensions: average quarterly wages among the sample of parolees, average job tenure among the sample of parolees, average quarterly wages among all employees at employers who hire parolees in the sample, and union coverage among all Michigan workers. I then use inverse propensity score weighting to compare the future criminal justice contact of parolees who are equally likely (based on demographic, human capital, and criminal history variables) to find work in a high-quality industry but who find work in industries that offer a different quality of employment. This results in an estimate of the effect of employment quality on future criminal justice contact net of a wide variety of controls that may jointly affect both employment quality and future criminal behavior.

I find that those whose first job after prison is in an industry that offers relatively high-quality employment are, in general, less likely to be arrested or recidivate during the two years after hire. The results of the models that account for differential selection into employment based on observable characteristics provide mixed evidence about whether high-quality employment is associated with reduced future criminal justice contact. When comparing those who find employment in the industries that offer the highest job quality—namely, manufacturing and transportation or warehousing—to those who find it in those that offer the lowest—namely, the employment services industry—the relationship between employment quality and the likelihood of being arrested in the two years after hire is more clear.

Notably, when comparing employment of varying quality to the counterfactual of not finding employment, I find evidence that high-quality, but not low-quality, employment is associated with a lower likelihood of returning to prison.

## POST-PRISON EMPLOYMENT AND FUTURE CRIMINAL JUSTICE CONTACT

Researchers studying the connection between post-prison employment and future criminal justice contact have offered a variety of mechanisms through which employment after incarceration should reduce the risk of recidivism. Most obviously, employment may reduce the risk of reoffending by reducing the motivation to commit crime for economic gain (Becker 1968; Freeman 1999). Similarly, anomic theories of crime (Merton 1938; Agnew 1985), which posit that individuals become motivated to commit crimes when they are unable to achieve socially normative goals using methods considered legitimate by wider society, suggest that employment reduces criminal behavior because it is a way to achieving economic and social goals. Employment may also act as an informal social control that prevents parolees from reoffending by inducing a sense that they have a stake in society (Toby 1957) or by providing continued interaction with individuals at work who are not in contact with the criminal justice system (Sampson and Laub 1993). Finally, employment may also reduce the risk of future criminal justice contact by providing a set of routine activities for workers, making it less likely that they will spend time in more criminogenic environments (Cohen and Felson 1979).

In particular, these mechanisms suggest that it is not just employment after release from prison but also the quality of the employment—as measured by objective markers such as earnings, job stability, and earnings growth—that should have a significant effect on future criminal justice contact. For one, highly paid employment may reduce immediate economic motivations for crime. Further, the social control perspective holds that jobs that provide longer tenure or offer regular, full-time work may be especially likely to inhibit future criminal justice contact, by leading to interdependence with professional social networks (Braithwaite 1989) and to the creation of new and durable routines to replace associations and activities from before prison that may have fostered criminal behavior (Crutchfield and Pitchford 1997). Robert Sampson and John Laub (1990, 611) highlight that it is not just employment but also “employment coupled with job stability, job commitment, and ties to work that should increase social control and, all else equal, lead to a reduction in criminal behavior.” Finally, employment that carries the potential for earnings growth over time is more likely to provide workers with a sense that they can achieve normative economic and social goals (Uggen 1999).^1^

However, Michael Gottfredson and Travis Hirschi (1990) argue that there is not a causal relationship between employment and future criminal justice contact. Specifically, they posit that both employment and crime are determined in part by the capacity for self-control. Individuals with low self-control will be less likely to find employment, much less high-quality employment, and will also be more likely to commit crimes. Under this logic, any effects of the quality of post-prison employment on future criminal justice contact would be spurious.^2^

Employment quality could also not affect recidivism if the pathway through which employment affects recidivism is decreased state surveillance. In *Poor Discipline,* Jonathan Simon (1993, 222) argues that parole officers often view any employment undertaken by parolees as a sign that they are on the right track and will thus be more lenient in supervising employed parolees or recommending parole revocations for violations such as occasional drug use. This, in turn, could cause employed parolees—no matter whether their job is high or low quality—to face lower risks of recidivism than those who are unemployed. If this difference in supervision is the main route through which employment affects recidivism, then we may not see a causal relationship between employment quality and future criminal justice contact.

One way that researchers have examined the connection between the quality of available employment and the propensity to commit crime is by using aggregate measures of local labor markets as a proxy for the quality of available employment. For example, Emilie Allan and Darrell Steffensmeier (1989) estimate that state-level rates of underemployment and low-wage employment are positively associated with arrest rates for young adults. Focusing on samples of released prisoners, Steven Raphael and David Weiman (2007) and Xia Wang, Daniel Mears, and William Bales (2010) find a small but statistically significant relationship between local unemployment rates and the probability of returning to custody. Similarly, Crystal Yang (2017) finds that prisoners who are released to counties with higher wages for workers without college degrees see lower rates of recidivism. Roberto Galbiati, Aurelie Ouss, and Arnaud Philippe (2017) and Kevin Schnepel (2018) examine the effect of industry-specific job openings on recidivism in France and California, respectively. Both articles find that the county-level creation of jobs in relatively high-quality industries—manufacturing in France, and construction and manufacturing in California—is associated with lower recidivism rates for inmates released within the county, although overall county-level job creation has no effect.

These results are strongly consistent with the thesis that it is the quality of employment, not merely being employed, that matters for future criminal justice contact. However, the cited studies generally have individual-level data on criminal justice system involvement but not on employment. They therefore do not allow us to disentangle the degree to which changes in local labor market conditions affect the likelihood of future criminal justice contact directly by affecting the quality of employment of recently released prisoners (that is, that increases in available jobs in the construction industry cause recently released prisoners to be more likely to work in the construction industry, and thus less likely to experience future criminal justice contact), rather than indirectly through other mechanisms (for example, whether increased economic opportunity makes a local environment less criminogenic in general).

Other research examining the connection between the quality of employment and future criminal justice contact has relied on individual-level data that include information on both employment and criminal justice contact after release.^3^ Christopher Uggen (1999) investigates the relationship between post-prison job quality and reoffending among a longitudinal sample of released prisoners using a sample selection model, which adjusts the relationship between job quality and reoffending among jobholders for unmeasured factors that jointly affect the propensity to enter employment and to reoffend. Based on this model, Uggen finds that job quality, measured using subjective job satisfaction scores from a nationally representative sample of workers, is a significantly negative predictor of recidivism, even after controlling for demographic characteristics and previous criminal history. However, the adjustment Uggen makes accounts for differential selection into any employment, not for differential selection into high-quality versus low-quality employment, and so does not necessarily disentangle any selection effects into high-quality versus low-quality employment from the effects of high-quality employment on recidivism. In addition, by controlling for job tenure and wages in predicting the effect of job quality on crime, Uggen’s analysis focuses on the effect of “the extraeconomic effects of job quality” (134). Although these extraeconomic dimensions of job quality are certainly important, this work does not test economic and anomic theories of crime, which explicitly suggest that the pecuniary rewards of employment are important predictors of future criminal justice contact.

Recent research by Anke Ramakers and colleagues (2017) uses propensity score techniques to account the probability that Dutch exprisoners who find employment in their first month after release do so in higher or lower occupational levels. These authors find a significantly negative effect of being in a higher (relative to a lower) occupational level on recidivism. However, given differences between labor market and criminal justice institutions between the Netherlands and the United States, generalizability of these findings to the United States context may be limited.

## DATA

I rely on longitudinal data on the employment outcomes of all prisoners paroled in the state of Michigan in 2003, collected from the Michigan Unemployment Insurance Agency and Workforce Development Agency. The benefit of unemployment insurance (UI) data is its comprehensive coverage: UI data capture virtually all formal employment undertaken in the state of Michigan for twenty-four quarters after the sampled individual’s release from prison. In each person-quarter, the data contain individual-level information on total wages earned from each Michigan-based employer, alongside employer-level information on the average quarterly wages paid to employees in the given quarter and the employer’s detailed six-digit North American Industry Classification (NAICS) Code.

I focus on the 10,794 individuals who are no older than fifty-five at the time of release. I estimate job quality using UI data from all these individuals; however, when I estimate the effect of employment quality on future criminal justice contact, I focus in particular on those who find employment, have not been arrested or returned to prison at any point between their sampled release from prison in 2003 and the end of the quarter in which they find such employment, and who do not have missing values for any covariates used in the estimation of the effect of job quality on future criminal justice contact.

### Job Quality

Because UI data do not include information about workers’ occupations, only the industries of their employers, I measure job quality at the level of the industry, using four distinct measures of job quality.

First, I compare average industry-level quarterly earnings of workers to average quarterly earnings in all other industries. Earnings are clearly a central component of job quality: higher earnings make it easier for workers to meet consumption needs and to grow savings. For parolees in particular, higher earnings may reduce recidivism by reducing economic motivations for crime. Calculating industry-level earnings within UI data is complicated by the fact that UI data do not have information on hourly wage rates or the number of hours that employees work within a quarter. Further, differences in job tenure between industries could affect calculations of average industry-level wages, both because higher-tenured workers earn more and because workers are more likely to work only for part of quarter in low-tenure industries. Thus, I compare industry-level wages between workers who work only at one job, are in their second quarter of employment at that job, and work at that job in the next quarter.

Second, I compare average industry-level employment tenure to average employment tenure in all other industries. Tenure is also an important dimension of job quality, reflecting stability of employment as well as the ability of workers to have careers within firms. Further, employers that provide greater employment stability may curb recidivism by providing a set of routine activities to replace those that led to imprisonment and creating deeper ties to individuals who are not in contact with the criminal justice system. I measure employment tenure as the number of consecutive quarters in a given employment spell with a given employer.

Third, I compare average industry-level quarterly wages per employee to average quarterly wages per employee in all other industries. This measure may reflect in part the potential for wage growth for employees who advance within the firm. Thus, being employed at a firm with higher quarterly wages per employee may provide workers who have just been released from prison with a sense that they are able to achieve normative economic and social goals, and therefore reduce the probability that they will return to prison. Harry Holzer and his colleagues (2011) use a similar measure—firm-level earnings effects—in using national UI data to compare how job quality varies by industry, arguing that this measure captures differences in firms’ contributions to pay due to capital holdings, compensating differentials, or human resources policies.^4^ Although this measure does not directly capture dimensions of job quality such as fringe benefits, growth opportunities, or safety, evidence suggests that these other dimensions are positively correlated with firm-level earnings effects (Andersson, Holzer, and Lane 2005; Hamermesh 1998). I calculate industry-level quarterly wages per employee using the average across the last quarter of all employment spells within the industry reported in the UI data.

Finally, I compare industry-level statewide union coverage rates to the average industry-level statewide union coverage rate. I calculate industry-level union coverage rates in the state of Michigan using data from 2003 through 2009 from the Current Population Survey, downloaded from CPS-IPUMS (Flood et al. 2018). It is unclear from UI data which parolees are covered by unions at their work. However, industry-level union coverage is likely to improve job quality for low-wage workers because non-union employers in highly unionized regions are compelled to raise job quality to forestall the threat of unionization. Research has found associations between region-industry union coverage and higher wages (Western and Rosenfeld 2011) and lower work hour volatility (LaBriola and Schneider 2019) for low-wage workers.

In sum, three of these four measures of job quality—average quarterly earnings, average employment tenure, and average firm-level quarterly wages per employee—are calculated from the sample of parolees in UI data and hence reflect the quality of the average job within each industry that sampled individuals might obtain after release from prison. The fourth measure—industry-level statewide unionization rates—reflects normative pressures that likely translate to better job quality for marginal workers. Although measuring job quality at the level of the industry has limitations (discussed in greater detail in the conclusion), the stark differences between industries in these measures of job quality strongly suggest that the measures meaningfully reflect how job quality varies between industries for individuals finding work after release from prison.

For each industry, I use two-tailed t-tests to determine whether average wages, average employment tenure, average quarterly wages per employee, and statewide union coverage are significantly greater than or less than average wages, average employment tenure, average quarterly wages per employee, and statewide union coverage in all other industries combined. I define an industry as offering high-quality employment if at least three out of four of these measures are significantly greater within the industry than in the rest of the sample, the fourth measure not being significantly lower. Conversely, I define an industry as offering low-quality employment if at least three out of four measures are significantly lower than in the rest of the sample, the fourth measure not being significantly higher. Within high-quality and low-quality industries, I also identify the highest-quality and lowest-quality industries that are above or below average in all four dimensions of job quality.

Table 1 tabulates the percentage of quarters worked by individuals in the sample by industry classification, along with average quarterly gross wages, average job tenure, average quarterly wages per employee, and statewide unionization rates by industry. Boldface entries in the columns measuring dimensions of employment quality indicate that the average value for a given industry is significantly larger than the average value in all other industries; italicized entries indicate that the average value for a given industry is significantly smaller than the average value in all other industries (a significance level of .05 is used for both).

The highest-quality industries in which sampled individuals find employment are manufacturing (NAICS = 31xxxx, 32xxxx, 33xxxx; 18.78 percent of quarters worked) and transportation and warehousing (NAICS = 48xxxx, 49xxxx; 1.89 percent of quarters worked); both industries offer above-average quarterly earnings, job tenure, firm-level quarterly earnings per employee, and state-level union coverage. Among common industries, construction (NAICS = 23xxxx; 9.19 percent of quarters worked) is also a high-quality industry, offering both above-average earnings, firm-level quarterly earnings per employee and state-level union coverage.

In contrast, employment services (NAICS = 5613xx; 19.66 percent of quarters worked in the sample) is the lowest-quality industry in which individuals paroled in Michigan in 2003 found employment, coming in below average in every measured dimension of employment quality. Industries classified as offering low-quality employment include limited service eating places (NAICS = 7222xx; 7.82 percent of quarters worked), full service restaurants (NAICS = 7221xx; 6.09 percent of quarters worked), and services to buildings and dwellings (NAICS = 5617xx; 3.69 percent of quarters worked).

I code the treatment variable as an indicator variable, equal to 1 if a sampled individual obtains their first employment after the sampled prison spell in a high-quality or highest-quality industry, and 0 if in a low-quality or lowest-quality industry. To more closely study the relationship between employment quality and future criminal justice contact, I focus on respondents who find post-prison employment before experiencing arrest or a return to prison. I assign individuals who have their first record of employment in a high-quality industry in the same quarter as they have their first record of employment in a low-quality industry a value of 1 for the treatment variable.

I also report results from a similarly constructed treatment variable where 1 indicates that a parolee finds employment in a highest-quality industry before getting arrested or returning to prison, and 0 indicates a lowest-quality industry. I use this treatment variable to test for the existence of any discernible effects of post-prison job quality on future criminal justice contact: if models that account for differential selection into job quality find no significant difference in future criminal justice contact between those who find the highest-quality employment and those who find the lowest-quality employment, no such effect likely exists.

Finally, I create four treatment variables to test for the effect of finding post-prison employment in an industry offering a given level of job quality relative to not finding employment. For each of four industry quality classifications (highest-quality, high-quality, low-quality, and lowest-quality), I set an indicator variable equal to 1 if a parolee finds employment in that industry category within one quarter of the quarter of release from prison, and 0 if not. As with other treatment variables, I drop observations when a parolee is arrested or has returned to prison within the time frame.

### Post-prison Criminal Justice Contact

I measure two types of criminal justice contact: arrests and returns to prison. Prison terms are certainly more consequential than arrests, and much of the literature on the effect of employment on future criminal justice contact focuses on recidivism as a dependent variable. Yet arrests are also an indicator of criminal behavior, and the various theories connecting employment quality to recidivism all suggest that high-quality employment should reduce criminal behavior more generally. I measure each of these types of criminal justice contact in each of the eight quarters after the quarter in which an individual finds employment in a high- or low-quality job. Data on arrests come from the Michigan State Police, and data on returns to prison come from the Michigan Department of Corrections.

**Figure 1.**
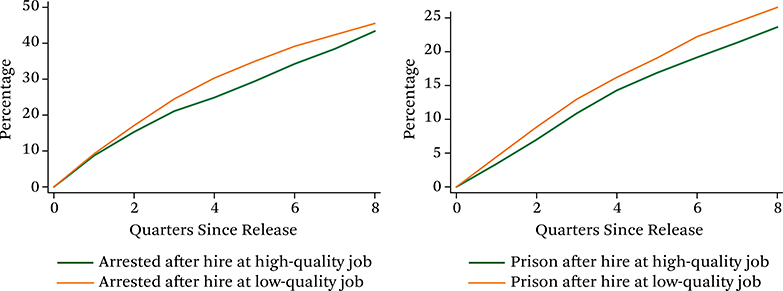
Cumulative Likelihood of Criminal Justice Contact After Finding Employment, by Employment Quality *Source:* Author’s compilation from data from the Michigan Unemployment Insurance Agency and the Michigan Workforce Development Agency.

Figure 1 shows the cumulative percentage of sampled parolees who are arrested or return to prison in the eight quarters after beginning work in a high- or low-quality industry. Criminal justice contact is fairly common for both groups, more than 40 percent of each set of workers experiencing an arrest within two years from starting employment and more than 25 percent of each set of workers experiencing a return to prison over this time frame. Sampled parolees who find high-quality employment are slightly less likely to experience arrest or a return to prison over the period, and the gap in criminal justice contact between those who find high- and low-quality employment widens over time.

### Other Covariates

Selection into a high-quality industry, relative to a low-quality industry, is a nonrandom process. Further, some of the same traits that predict this selection process are also likely to predict whether an individual is likely to recidivate. Therefore, to more closely estimate the causal effect of finding employment after release from prison in a high-quality industry (relative to a low-quality one), it is necessary to control for factors that could affect both the quality of industry in which parolees find employment and the likelihood that parolees experience future criminal justice contact.

I include as covariates several variables that measure parolees’ demographic and human capital characteristics—age at time of release, sex, race (operationalized as white or black),^5^ an indicator for being married at time of release, whether an individual has a high school degree or GED, and the logged maximum quarterly earnings recorded in the Michigan UI data between 1997 and the time of release. I also include as covariates the quarterly unemployment rate at the time that the parolee first found employment in the county in which the parolee first lived after release from prison (collected from the Michigan Department of Labor and Economic Growth), and the quarter after release from prison in which the respondent found employment.

Finally, I include a host of covariates collected by the Michigan Department of Corrections that reflect both parolees’ exposure to the criminal justice system through the sampled release from prison and factors used in previous research to predict post-prison employment and criminal justice outcomes (Uggen 1999). Notably, given that Josh Seim and David Harding (2020) find that parole supervision may impel parolees into obtaining employment, I control for a proxy of the intensity of parole supervision: whether a parolee is subject to electronic monitoring.

Table 2 lists all model covariates and displays their mean values within the sample by employment quality. Parolees who found employment in high-quality industries are older, had higher pre-prison earnings, and are more likely to be white, male, and married than their counterparts in low-quality industries. Although those who found employment in low-quality industries committed more frequent misconducts during their sampled prison spell, these groups surprisingly do not otherwise appear to differ in the levels of variables reflecting their criminal justice history. The final analysis sample of those who have nonmissing values on all covariates consists of 1,026 individuals who found employment in high-quality industries and 2,529 who found employment in low-quality industries.

## METHODS

I first estimate the naïve treatment effect of employment quality on each of the measures of future criminal justice contact using a series of linear probability regression models:^6^

(1) $$Prob\left(Y_{it}\right)=\alpha +\beta_{1}HQ_{i}+\varepsilon_{i},$$

where *Y*_*it*_ is an indicator variable equal to 1 if individual *i* has experienced a given form of criminal justice contact in the *t* quarters since finding high- or low-quality employment and 0 if not, *HQ*_*i*_ is the treatment variable described, and *ε*_*i*_ is a standard error term. These models estimate what the effect of post-prison employment quality would be on future criminal justice contact if selection into post-prison employment quality were random, and essentially capture the difference in probability of experiencing future criminal justice contact between those who find high-quality and low-quality employment. Negative values of β_*1*_ indicate that those with high-quality employment are less likely to experience a given form of future criminal justice contact, whereas positive values indicate that those with high-quality employment are more likely.

Next, I compare these naïve estimates of effects of employment quality on future criminal justice contact to estimates that use inverse propensity score weighting (IPW) (see, for example, Morgan and Winship 2015, 226–66) to account for differential selection into employment of differing quality. To do so, I first estimate the propensity score—here, the probability that an individual in the final analysis sample finds employment in a high-quality industry—using the following logistic model:

(2) $$log\left(\frac{Prob\left(HQ_{i}\right)}{1-Prob\left(HQ_{i}\right)}\right)\alpha +\beta_{1}X_{i}+\varepsilon_{i},$$

where *X*_*I*_ is the vector of covariates listed for individual *i* and other variables are as previously defined. Then, I use the predicted propensity scores from the above regression to assign each individual *i* weights as follows:

(3) $$w_{i}=\frac{1}{Prob\left(HQ_{i}\right)}\;\text{if}\;i\;\text{finds}\;\text{high-quality}\;\text{employment}\;\;\;\;w_{i}=\frac{1}{1-Prob\left(HQ_{i}\right)}\;\text{if}\;i\;\text{finds}\;\text{low-quality}\;\text{employment}.$$

Here, observations are weighted by the inverse of the probability of receiving the treatment (of high-quality or low-quality employment) they actually received. This weighting gives more weight to individuals who find high-quality employment and are more similar to those who find low-quality employment on covariates included in the propensity score model, and vice versa. In essence, this creates a pseudo-population where post-prison employment quality is uncorrelated with the covariates that affect both employment quality and future criminal justice contact.^7^

Finally, I include these weights in a weighted linear probability model predicting future criminal justice contact as a function of job quality and covariates listed above:

(4) $$Prob\left(Y_{it}\right)=\alpha +\beta_{1}HQ_{i}+\beta_{2}X_{i}+\varepsilon_{i}.$$

I specify this model to have robust standard errors. The inclusion of covariates in both the model predicting employment quality and the model predicting future criminal justice contact—known as “doubly-robust” regression (Morgan and Winship 2015, 234–37)—increases the likelihood that this modeling process obtains an unbiased effect estimate, because if either model is correctly specified, the estimated treatment effect will be unbiased.

I estimate the naïve and IPW models for two measures of future criminal justice contact—being arrested after finding employment and returning to prison after finding employment. For each measure, I estimate the effect on the cumulative probability of experiencing criminal justice contact between the time of finding employment and one to eight quarters afterward. Negative values of *β*_1_ indicate that high-quality employment leads to lower risk of criminal justice contact; positive values of *β*_1_ indicate that high-quality employment leads to higher risk of criminal justice contact.

I also replicate the analysis using a treatment variable that compares future criminal justice contact of parolees whose first post-prison employment is in a highest-quality industry (manufacturing and transportation-warehousing) to their counterparts in the lowest-quality industry. Finally, I use the same method to estimate the respective effects of finding employment in a highest-quality, high-quality, low-quality, and lowest-quality industry within the first quarter after the quarter of release from prison, relative to not finding employment in that time frame.

## RESULTS

I first discuss estimates of the effect of employment quality on future criminal justice contact. I then turn to discussing how estimates of the relationship between employment and future criminal justice contact vary by employment quality.

### Effects of Employment Quality on Future Criminal Justice Contact

**Figure 2.**
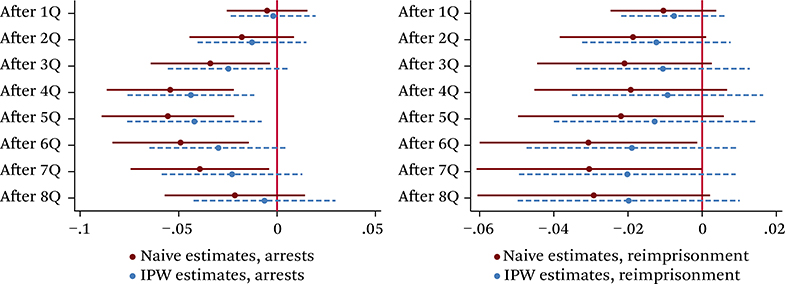
Effect of High-Quality Versus Low-Quality Employment on Future Criminal Justice Contact *Source:* Author’s compilation from data from the Michigan Unemployment Insurance Agency and the Michigan Workforce Development Agency. *Note:* These figures represent estimates of the effect of finding employment after release from prison in an industry that offers high-quality employment (relative to finding employment in an industry that offers low-quality employment) on the cumulative likelihood of experiencing an arrest (left panel) or returning to prison (right panel) in each of the eight quarters after finding employment. High-quality industries include manufacturing, transportation and warehousing, construction, educational services, and mining; low-quality industries include services to buildings and dwellings, employment services, arts-entertainment-recreation, accommodation and food services, and other services. Estimates are expressed in percentage points, with negative values indicating reduced future criminal justice contact for those who find high-quality employment. The estimates with solid-line confidence intervals represent the naïve difference in future criminal justice contact between those who find high- and low-quality employment. The estimates with dashed-line confidence intervals represent the estimated difference in future criminal justice contact between those who find high- and low-quality employment that accounts for differential selection into employment quality using inverse propensity score weighting.

Figure 2 presents both the naïve estimates (solid line confidence intervals) and the estimates from the IPW procedure (dashed line confidence intervals) of the treatment effect of finding high-quality post-prison employment (relative to low-quality) on future criminal justice contact. The left panel shows effects for the outcome of being arrested after finding employment; the right panel shows effects for the outcome of returning to prison after finding employment. The magnitude of the estimated effect on the probability of being arrested is shown on the x-axis. Estimated effects are ordered vertically by time such that the estimated effect on the probability of being arrested or returning to prison in the first quarter after finding employment is at the top of the figure and the estimated effect for within eight quarters is at the bottom.

The naïve estimates of the effect of employment quality on future criminal justice contact restate the findings in figure 1: those who find high-quality employment are less likely, and often significantly less likely, to experience future criminal justice contact in the quarters after starting employment. However, the IPW estimates, which account for differential selection into employment quality based on observable characteristics, are lower and less often significantly different from zero. For the outcome of being arrested, the cumulative risk is significantly lower for those who find employment in high-quality industries through the fourth and fifth quarters after finding employment; however, for the outcome of returning to prison, no significant causal effect of employment quality is evident. Overall, the effect of finding employment in a high-quality relative to a low-quality industry on future criminal justice contact appears to be positive, but the estimates are decidedly mixed.

**Figure 3.**
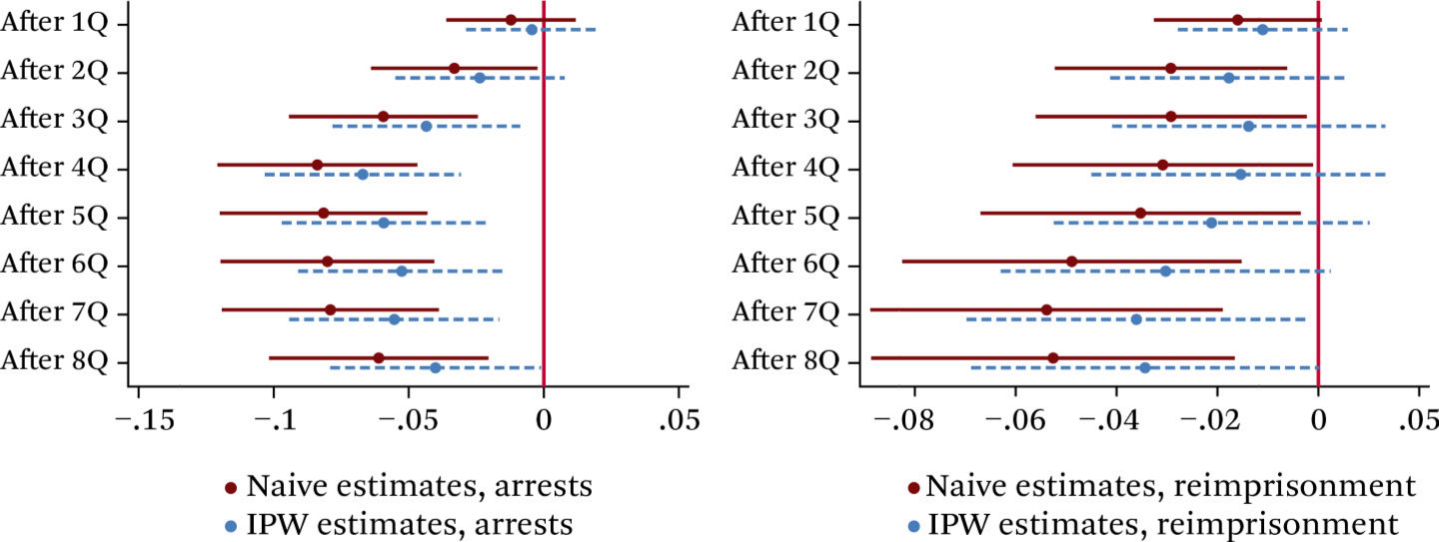
Effect of Highest-Quality Versus Lowest-Quality Employment on Future Criminal Justice Contact *Source:* Author’s compilation from data from the Michigan Unemployment Insurance Agency and the Michigan Workforce Development Agency. *Note:* These figures represent estimates of the effect of finding employment after release from prison in an industry that offers highest-quality employment (relative to finding employment in an industry that offers lowest-quality employment) on the cumulative likelihood of experiencing an arrest (left panel) or returning to prison (right panel) in each of the eight quarters after finding employment. Highest-quality industries include manufacturing and transportation and warehousing; lowest-quality industries include employment services. Estimates are expressed in percentage points, with negative values indicating reduced future criminal justice contact for those who find highest-quality employment. The estimates with solid-line 95 percent confidence intervals represent the naïve difference in future criminal justice contact between those who find highest- and lowest-quality employment. The estimates with dashed-line 95 percent confidence intervals represent the estimated difference in future criminal justice contact between those who find highest- and lowest-quality employment that accounts for differential selection into employment quality using inverse propensity score weighting.

Figure 3 presents the results of the naïve and IPW estimates of the effect of finding employment in one of the highest-quality industries—manufacturing, transportation, and warehousing—relative to finding employment in the lowest-quality industry—employment services—on future criminal justice contact. If employment quality does in fact reduce future criminal justice contact, the effects should be most apparent when comparing the highest- and lowest-quality industries.

Here we do see a more consistent effect of employment quality on future criminal justice contact, especially for arrests. After accounting for observable characteristics, those who find employment in the highest-quality industries are just over 4 percentage points less likely to be arrested between starting employment and eight quarters afterward; this corresponds to a roughly 10 percent decrease in the likelihood of being arrested over the period. Similarly, those who find employment in the highest-quality industries are almost 4 percentage points less likely to return to prison in the eight quarters after starting employment than those who find employment in employment services; this translates to about a 13 percent decrease in the likelihood of returning to prison.

### Effects of Employment on Future Criminal Justice Contact, by Employment Quality

**Figure 4.**
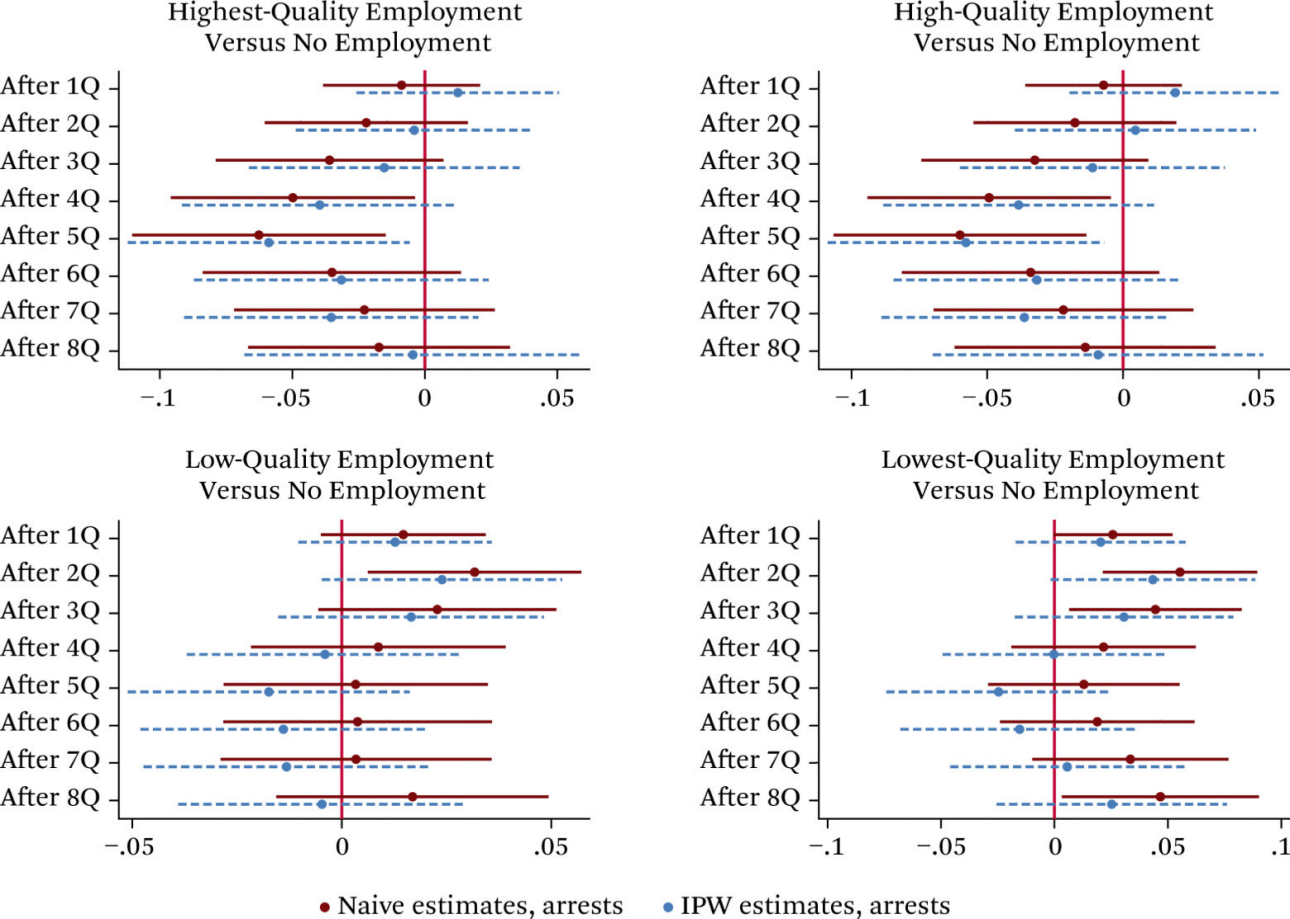
Effect of Employment on Future Arrests, by Employment Quality *Source:* Author’s compilation from data from the Michigan Unemployment Insurance Agency and the Michigan Workforce Development Agency. *Note:* These figures represent estimates of the effect of finding employment within the first quarter after release from prison in industries that offer varying qualities of employment (relative to not finding employment in this time) on the cumulative likelihood of experiencing an arrest in each of the eight quarters after this time. The estimates with solid-line 95 percent confidence intervals represent the naïve difference in future arrests between those who find employment within the first quarter after release from prison and those who do not find employment. The estimates with dashed-line 95 percent confidence intervals) represent the estimated difference in future arrests that accounts for differential selection into employment of varying quality using inverse propensity score weighting.

**Figure 5.**
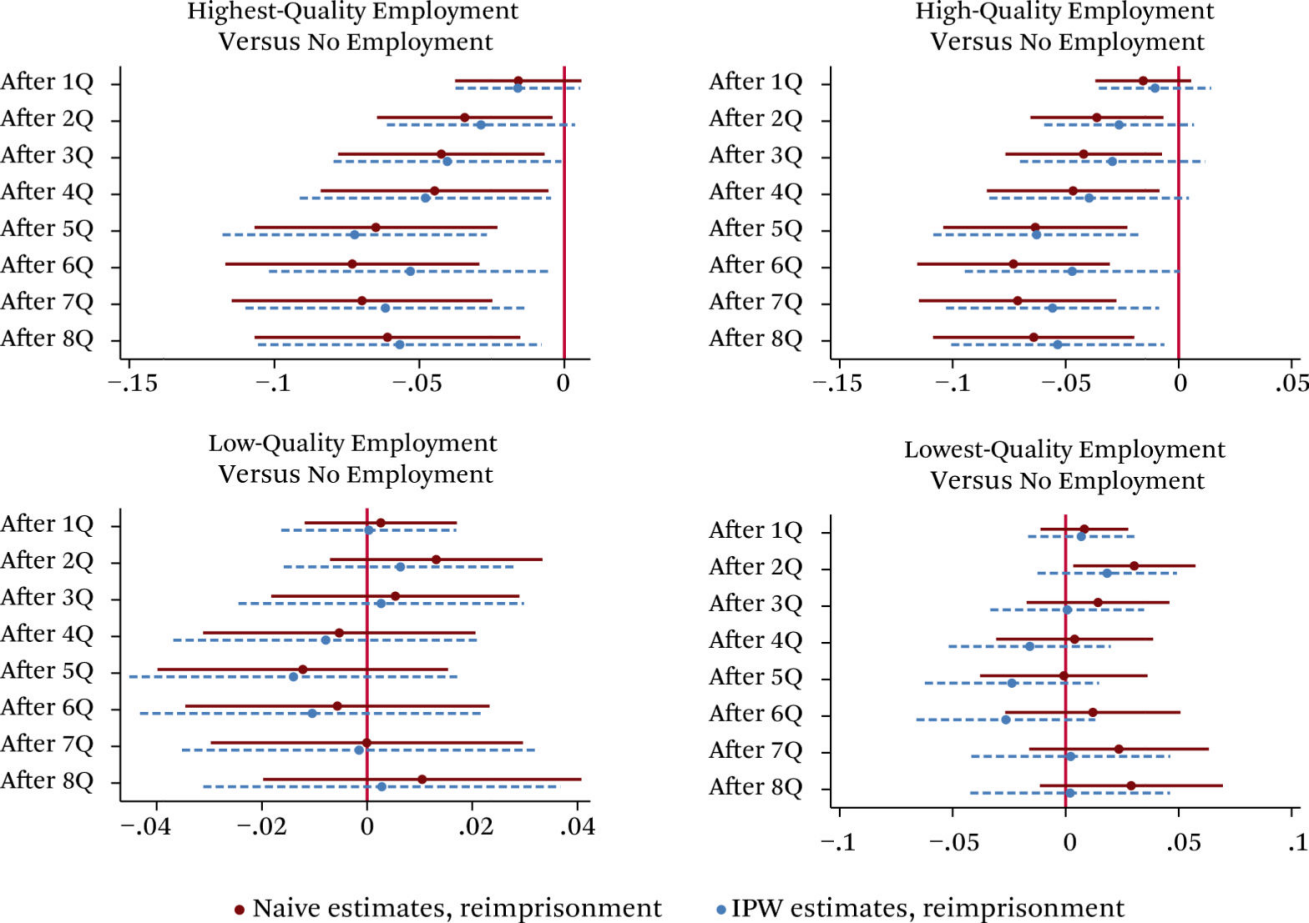
Effect of Employment on Future Reimprisonment, by Employment Quality *Source:* Author’s compilation from data from the Michigan Unemployment Insurance Agency and the Michigan Workforce Development Agency. *Note:* These figures represent estimates of the effect of finding employment within the first quarter after release from prison in industries that offer varying qualities of employment (relative to not finding employment in this time) on the cumulative likelihood of returning to prison in each of the eight quarters after this time. The estimates with solid-line 95 percent confidence intervals represent the naïve difference in returning to prison between those who find employment within the first quarter after release from prison and those who do not. The estimates with dashed-line 95 percent confidence intervals represent the estimated difference in returning to prison that accounts for differential selection into employment of varying quality using inverse propensity score weighting.

Figure 4 presents naïve and IPW estimates of the effect of finding employment by the first quarter after release from prison (relative to not doing so) on the cumulative risk of arrest in the eight quarters afterwards, by employment quality. Figure 5 presents analogous estimates for the outcome of the cumulative risk of returning to prison.

We see in figure 4 that employment in itself does not appear to have a robust effect on reducing future arrests, no matter the quality of employment. Interestingly, those who find employment in low-quality industries actually appear to be more likely to be arrested in the first two or three quarters after finding employment than those who do not find employment at all. Figure 5, however, shows strong evidence that high-quality employment reduces the cumulative risk of returning to prison in the eight quarters after beginning employment relative to not finding employment. Finding low-quality employment, on the other hand, appears to have no such effect.

### DISCUSSION

Social scientists have put forth several causal explanations for criminal behavior, including economic motivation, anomic isolation, lack of social control, and lack of routine activities. Each of these theories suggests that, for those who have had previous criminal justice contact, not just employment but employment quality should matter for future criminal justice contact. Although previous work has found an effect of employment quality on recidivism in the context of the United States, the question of whether this effect can be accounted for by selection into various types of employment is unanswered. Further, this work focused on extraeconomic components of job quality, although economic aspects of job quality are also thought to be important for reducing motivations for crime.

I use matching techniques to compare otherwise similar parolees who find employment in industries characterized by varying levels of employment quality, as defined by average earnings, job tenure, firm-wide earnings per employee, and state-level union coverage. Some evidence indicates that high-quality employment reduces the risk of future criminal justice contact relative to lowest-quality employment, though this effect is most apparent when comparing the industries that offer the best employment quality to the industry that offers the worst.

I also analyze how employment in industries of varying quality affects the future likelihood of arrest or reimprisonment relative to those who do not find employment. I find that securing employment in high-quality industries reduces the risk of future criminal justice contact, but that in low-quality industries it does not. This may imply that, due to the increasing precarity of work in the United States (Kalleberg 2011), much of the employment available to parolees may not be able to provide economic benefits or social integration that are thought to link post-prison employment to reduced criminal justice contact. These results are consistent with the findings of Seim and Harding (2020), who use the same data as in this article to show that, among both those on parole and those who have been discharged from parole, employment appears to have negligible effects on recidivism. The most obvious explanation for these results is that most individuals who find work after prison do so in relatively low-quality industries: the counts in table 2 suggest that parolees are roughly two and a half times as likely to find low-quality employment as high-quality employment after release from prison. Further, even high-quality employment does not forestall all future criminal justice contact, and many of the estimates of the effect of high-quality employment on criminal justice contact have confidence intervals that overlap with zero.

These findings should be qualified in several ways. Perhaps the most salient limitation is that the UI data do not include information about workers’ occupations, which would likely give more precise information about workers’ job quality than is available at the industry level. If there is heterogeneity in job quality within industries classified here as high-quality or low-quality, then this would attenuate the estimates of the effect of job quality on future criminal justice contact toward zero. Given the limitations of this data, it is impossible to determine the extent to which this is the case. However, despite variation in job quality within industries in general, those who have been to prison are likely to mostly be able to find employment in jobs that are among the lowest quality within industries, given their poor job prospects overall (Western 2006). This would imply relatively little within-industry heterogeneity in job quality in this sample. Future research that has access to detailed data on parolees’ trajectories of occupations and criminal justice contact after release from prison could help test the extent to which post-prison occupational quality varies within industries.

A second qualification is that UI data do not capture informal employment, which some surveys have found is as common as formal employment within the first year of release from prison (Visher et al. 2008). Informal employment is likely to be of worse quality than the formal employment along dimensions of wages, job tenure, wage growth, and worker protections (Nightingale and Wandner 2011), and is likely associated with less structure than formal employment is. This implies that informal employment may be less likely than formal employment to prevent future criminal justice contact. However, with this data, this proposition cannot be tested.

Third, and relatedly, this study focuses on only a small sample of the population of those who have been released from prison. Most notably, formal employment is generally rare in this sample: fewer than one in three parolees are employed in the formal labor market in any given quarter after release (author’s calculations). These low rates are similar to those found in previous research (Pettit and Lyons 2007; Sabol 2007), and are likely caused by several processes, including stigma against those with a criminal justice history (Pager 2003), state-level “hidden sentences” that hinder employment (Warner, Kaiser, and Houle 2020), and monetary sanctions, which interfere with the ability to maintain employment in several ways (Cadigan and Kirk 2020). Because this study focuses on parolees who are most employable, it cannot speak to how the future criminal justice contact of other parolees would be affected by finding employment of varying quality.

Fourth, it is possible that the relationship found here between employment quality and future criminal justice contact could result from those who are ready to desist from crime pursuing higher-quality employment as a “signal” (Bushway and Apel 2012) to others of their intentions to desist. In this scenario, those who search for high-quality employment may be disproportionately less likely to experience criminal justice contact, no matter their actual post-prison employment trajectory. Although this explanation cannot be completely ruled out with the data in this study, I am able to use a wide set of demographic, human capital, and criminal justice-related individual controls to account for selection into employment quality.

Finally, this study follows only one cohort of parolees who exit prison into a particular social and economic context, and so the external validity of these findings is limited. It would be useful to conduct similar analyses of the effect of job quality on recidivism in different times and places to build a more robust body of evidence about this relationship.

Although I find that employment quality reduces future criminal justice contact, it should still be emphasized that sampled individuals experience relatively short job tenures across all industries, and the difference in job tenure between high- and low-quality industries is often less than one quarter. Thus, any protective effects of relatively high-quality employment may be unlikely to last. Although those who have been to prison may be generally less likely than others to find and maintain stable, well-paying employment, it also seems probable that parolees are especially likely to experience precarious work, given that work has become more precarious generally in the United States (Kalleberg 2011), especially for workers of lower socioeconomic status. It is therefore plausible that increases in job quality and worker protection for all low-wage workers may facilitate the ability of those who have been to prison to maintain stable, well-paying employment, which may then have longer-lasting effects on future criminal justice contact.

Such a shift in the focus of research and policy interventions—from recidivism to the broader environment to which parolees return to after release from prison—echoes recent commentaries (Butts and Schiraldi 2018) that emphasize that recidivism is not reducible to the behavior of individuals alone. Recidivism is contingent on law enforcement’s becoming aware of illegal acts committed by those who have committed crimes before; because state surveillance is more common in neighborhoods where poor people and people of color live, the risk of recidivism among otherwise equal individuals is not distributed equally. Although these criminal justice system inequalities have deservedly received increased attention as a site of intervention in recent years, labor market institutions are also important sites of intervention—not merely for the effects they may have on recidivism, but also for their potential impact on parolees’ ability to positively participate in the social life of their community.

## Figures and Tables

**Table 1.** Within-Sample Measures of Employment Quality, by Industry Classification

Industry Name	NAICS Prefix	Percentage Quarters Worked	Average Quarterly Earnings	Average Job Tenure (Quarters)	Firm Average Quarterly Earnings	State-Level Union Coverage

All industries		100%	5,366	2.03	4,361	21.6%
**Highest-quality industries**						
Manufacturing	31–33	18.78	**6,741**	**2.65**	**7,831**	**26.4**
Transportation and warehousing	48–49	1.89	**6,832**	**2.48**	**6,389**	**42.9**
**High-quality industries**						
Construction	23	9.19	**7,407**	2.06	**6,724**	**27.2**
Educational services	61	0.81	6,132	**2.52**	**8,333**	**52.7**
Mining	21	0.14	**8,309**	2.03	**9,127**	**44.7**
**Medium-quality industries**						
Retail trade	44–45	7.47	5,299	**2.13**	**4,759**	*10.9*
Health care and social assistance	62	5.16	*2,837*	**2.77**	*3,181*	*15.3*
Wholesale trade	42	3.18	**6,429**	**2.7**	**7,913**	*9.5*
Automotive repair and maintenance	8111	2.46	5,302	**2.4**	4,299	*2.4*
Other administrative and support services	56	2.16	5,496	*1.82*	4,297	*5.5*
Professional, scientific, and technical services	54	1.96	**7,826**	2.11	**5,637**	*2.9*
Missing NAICS code	.	1.25	6,167	*1.57*	4,254	NA
Real estate and rental and leasing	53	0.91	6,052	2.21	**4,947**	NA
Public administration	92	0.56	6,456	2.25	**10,360**	**55.5**
Agriculture, forestry, fishing, and hunting	11	0.55	6,049	*1.75*	3,270	*2.9*
Information	51	0.50	**5,302**	**2.36**	**6,219**	*18.1*
Finance and insurance	52	0.45	**6,904**	**2.59**	**6,475**	*5.3*
Management of companies and enterprises	55	0.12	5,780	2.41	**7,381**	*0.0*
Utilities	22	0.01		3	**17,230**	**46.7**
**Low-quality industries**						
Limited service eating places	7222	7.82	*2,852*	1.98	*2,241*	*2.0*
Full service restaurants	7221	6.09	*3,820*	2.03	*2,671*	*2.0*
Services to buildings and dwellings	5617	3.69	4,976	*1.92*	*3,660*	*6.3*
Other accommodation and foodservices	72	2.73	*4,067*	1.99	*2,837*	*6.0*
Other services	81	1.56	*4,434*	2.06	*3,684*	*4.6*
Arts, entertainment, and recreation	71	0.90	*4,260*	1.99	*2,908*	*10.7*
**Lowest-quality industries**						
Employment services	5613	19.66	*4,205*	*1.59*	*2,677*	*4.2*

*Source:* Author’s compilation from data from the Michigan Unemployment Insurance Agency, the Michigan Workforce Development Agency, and the 2003–2009 Current Population Survey (Flood et al. 2018).

*Note:* Boldface entries indicate significantly higher job quality than in other industries (*p* < .05, two-tailed test).

Italicized entries indicate significantly lower job quality than in other industries. Quarterly gross wages calculated among all workers in the sample working in the second consecutive quarter working for an employer, and who are still working for that employer in the next quarter. Average quarterly gross wages per employee calculated during the last quarter working for an employer.

**Table 2.** Means of Model Covariates, by Post-prison Employment Quality

	Low-Quality Employment	High-Quality Employment

Age at release from prison	33.3	35.1***
Percentage white	45.0	54.4***
Percentage female	10.1	5***
Percentage married	12.4	15.7**
Percentage with high school degree or GED	57.8	60.2
Logged max quarterly pre-prison wages	5.4	5.7*
Number of prior arrests	4.9	5.2
Percentage with conviction for violent offense	47.5	47.6
Percentage with conviction for theft	45.2	44.3
Percentage with conviction for drug offense	31.8	29.9
Percentage with conviction for behavioral offense	24.9	26.7
Number of years in prison spell	3.3	3.4
Maximum management level during prison spell	2.7	2.6
Misconducts per year during prison spell	0.8	0.6***
Percentage time in solitary during prison spell	1.1	0.8
Percentage classified as sex offender	8.3	8.1
Percentage known to have mental illness	18.6	18.0
Percentage with known substance abuse history	43.9	43.4
Percentage subject to post-prison electronic monitoring	8.9	11.3*
Unemployment rate in county to which parolee released, in quarter of release	7.4	7.5
Time to finding post-prison employment (quarters after release)	2.5	2.5
N	2,529	1,026

*Source:* Author’s compilation from data from the Michigan Unemployment Insurance Agency and the Michigan Workforce Development Agency.

* *p* < .05

** *p* < .01

*** *p* < .001

## Footnotes

1. Although this study focuses on job quality as measured by objective measures, it is also plausible that subjective markers of job quality, such as the desirability of an occupation (Uggen 1999) or a sense that a job is significant or allows the employee to do what they do best (Wadsworth 2006), lead to reduced future criminal justice contact for those who have been to prison. Jobs experienced subjectively as high quality may give these workers additional motivation to desist from crime so as to not jeopardize their employment. Further, routine activities and social control theories suggest that subjective job quality could lead to reduced future criminal justice contact if subjective job quality increases job tenure.

2. The claim that employment does not affect future criminal justice contact is supported by the meta-analysis conducted by Christy Visher, Sara Debus, and Jennifer Yahner (2008), which finds that experimental assignment to employment programs does not reduce recidivism. However, these results do not necessarily speak to how different types of employment may differentially affect recidivism. If, for example, these programs do not place those who have been to prison in high-quality jobs, and only high-quality jobs affect future criminal justice contact, we could still observe that employment programs do not affect recidivism.

3. Although this study focuses on the effect of job quality on future criminal justice contact for those who have been recently released from prison, the literature on how job quality is associated with criminal behavior among a broader population of young adults is sizeable. For example, Jeff Grogger (1998) finds that wages are negatively associated with criminal activity among young adults, whereas Tim Wadsworth (2006) finds that subjective measures of job quality are more important than objective measures in predicting self-reported crime. Other work examines a population of those who have just been admitted to prison and considers how job quality co-varies with self-reported criminal activity in the time before prison admittance (Apel and Horney 2017).

4. In results available on request, I test the robustness of this article’s findings to measuring the quality of industries via industry-level average firm earnings effects (calculated from Holzer et al. 2011, table 2.1). Using this measure to classify industry quality reveals largely similar results to those reported in this article—higher-quality jobs reduce future criminal justice contact. However, the industry groupings used in the study by Harry Holzer and his colleagues (2011) are not granular enough to capture the industry of employment services (temporary help agencies), which offers the worst job quality to parolees in the Michigan data set. These jobs are contained in the administrative and support services category, which Holzer and his colleagues do not rate as low in quality as other industries. Further, within-sample measures of industry-level job quality are more likely to capture parolees’ experience of job quality than are national measures of industry-level job quality. Thus, I chiefly rely on these within-sample measures of industry-level job quality.

5. The race of 98 percent of parolees captured in the data is white or black. The analysis focuses on these individuals because the small number of individuals of another race in the dataset does not allow for strong inference on the effects of job quality within this group. Analyses that dichotomize individuals’ race as white or nonwhite produce similar results.

6. I use linear probability models rather than logistic regression models to facilitate the comparison of coefficients across models, because doing so using the results of logistic regression models is inadvisable (Mood 2010).

7. For weighting to remove the association between covariates that affect the outcome and the treatment, the distribution of covariates must not vary between the weighted treatment and weighted control groups. In each model, I implement the test of Kosuke Imai and Marc Ratkovic (2014) to check whether covariates are balanced between treatment and control groups, and in all cases do not reject the null hypothesis that the propensity score model balances covariates between groups.
